# Integrating teacher data literacy with TPACK: A self-report study based on a novel framework for teachers' professional development

**DOI:** 10.3389/fpsyg.2022.966575

**Published:** 2022-09-29

**Authors:** Yulu Cui, Hai Zhang

**Affiliations:** ^1^School of Information Science and Technology, Northeast Normal University, Changchun, China; ^2^School of Media Science/School of Journalism, Northeast Normal University, Changchun, China

**Keywords:** teacher data literacy, framework, technological pedagogical content knowledge, teachers' professional development, ORID

## Abstract

While teachers' knowledge is widely viewed as a key aspect of professional development in the new era, little research attention has been paid to one of its key components: teacher data literacy. Accordingly, this study aimed to combine teacher data literacy with TPACK (technological pedagogical content knowledge), a widely-used framework for understanding and assessing teachers' knowledge. We first used qualitative methods to develop this integrated framework, then distributed a quantitative self-report survey based on the framework to teachers, and analyzed the resulting data. The qualitative phase highlighted five types of teachers' knowledge required in an integrated core knowledge system incorporating data literacy and provided insights for reflecting on teaching and learning in smart learning environments. The quantitative analysis of data from the TDL-TPACK questionnaire indicated that most teachers were competent practitioners but had some areas for improvement. Experienced teachers in their 30s and 40s performed at higher levels, while some of those aged over 50 displayed incremental decreases in performance. Other factors such as the age, experience, academic qualifications, and role of teachers may affect different aspects of their knowledge, including their data literacy. The research findings provide useful insights for additional teacher training and development programs in the context of smart education.

## Introduction

Teachers are often described as experts whose knowledge and skills benefit students in the classroom environment (Yenen, 2021). However, emerging technologies and policy-driven changes require their knowledge, skills, and literacy to be updated continuously. In particular, smart education generates large amounts of data with great potential for better teaching and more effective professional development (Cui and Zhang, 2021). The widespread use of learning technologies, tools, and platforms during the COVID-19 pandemic also indicated that data literacy would enable teachers to generate better experiences for their students. This confirmed that in earlier research evidence-based instruction results in greater academic growth (Jung et al., 2018; Gesel et al., 2021). Accordingly, the knowledge and skills required to meet the complex and growing demands of educational reforms in the current era are particularly important for teachers (Yenen, 2021).

Adoniou indicated that teachers' knowledge must be continuously updated (Adoniou, 2015), however data usage is still a complex area: transforming data into information and information into meaningful decisions requires a wide range of knowledge and skills (Mandinach and Gummer, 2016b; Van Gasse et al., 2021). Among the three factors influencing teachers' knowledge identified by a recent 52-item survey was data-based decision-making (DDM; Malatesha Joshi and Wijekumar, 2019). However, teachers performed less well in DDM compared to the other two factors, demonstrating the difficulty many teachers encounter when trying to integrate data-related knowledge and skills into their core professional capacities.

Previous research has identified significant gaps in teacher data literacy (TDL). For instance, most of the teachers in Filderman et al.'s (2021) study displayed difficulties in locating, comprehending, and interpreting data. While teachers have long worked with data, their roles have traditionally positioned them more passively as evaluators and implementers of educational interventions (Xin, 2021). However, the increasing amounts of data—such as student ranking and examination scores—available in smart learning environments pose a challenge to teachers. One common coping strategy is avoidance: teachers often limit how they use data to simple performance evaluations of students while seldom applying data to the tasks of improving teaching and securing long-term student development (Gelderblom et al., 2016). Many teachers can only understand simple data presentations and struggle to interpret complex presented data such as box plots; up to one-third of teachers in one study read data erroneously (Pierce et al., 2014). While teachers are concerned with data-driven educational interventions, they significantly lack data literacy skills (Reeves and Chiang, 2018) related to data mining and use. A key issue is that TDL rarely features in teacher training programs, so educators must strive to teach themselves how to integrate data literacy with pedagogy (Mandinach and Schildkamp, 2021).

Although models such as the technological pedagogical content knowledge framework (TPACK; Falloon, 2020) are effective, they generally overlook the use of data in teaching and learning. Voogt and McKenney (2017) noted that TPACK, the integration of technology into educational practice, systematizes a complex and emergent process. Similarly, the effectiveness of information and communications technology (ICT) applications does not automatically transfer to natural educational settings. Teachers, especially novice educators, often report a lack of foundational knowledge in data-related knowledge and skills (Dunlap and Piro, 2016). Accordingly, the TPACK framework is currently unable to integrate data-related knowledge and skills, an area that would undoubtedly improve the professional development of teachers.

Educational researchers emphasize the need to integrate pedagogic content knowledge (PCK) with data literacy, but the close linkages between content knowledge and data use are rarely acknowledged (Mandinach et al., 2015b). This impacts the professional development of teachers, since data interpretation, decision-making, and practical action are important components of PCK (Haiyan, 2021). Researchers also underline that teachers must reflect on how to integrate the knowledge of teaching, content, and data into their practice (Jinliang and Baozhen, 2015). In other words, data literacy should be integrated into the general concept of teacher competence to improve professional development outcomes at all teaching levels (Raffaghelli, 2019). This is even more crucial for pre-service teachers because data competencies must be linked to their pedagogical content knowledge to optimize their skills as instructors (Mandinach and Gummer, 2016b). Hence, further investigation must establish the relationships between data skills and PCK to refashion training that integrates data literacy into future TPD curriculums (Mandinach and Schildkamp, 2021). Focusing on this study, our key areas of inquiry were to investigate how TDL might be integrated into teachers' core knowledge (TPACK), to determine the current state of data literacy among teachers, and to reflect on how TDL might feature in future professional development activities.

Considering the above needs, this research aimed to (1) integrate TDL with TPACK to form a new framework and (2) apply it to promote professional development initiatives in future training programs. To achieve the first aim of this mixed-methods research, the objective–reflective–interpretive–decisional (ORID) method (qualitative) was deployed to obtain the consensus of participants. The second goal was supported by a large-scale self-report questionnaire survey (quantitative) to explore the current state of teachers' professional knowledge.

## Theoretical background

### Teacher data literacy

Literacy is a key skill and a widely used measure of a population's educational level. In recent years, global literacy levels have risen tremendously alongside major improvements in basic education (Roser and Ortiz-Ospina, 2016). However, this raises the question of how we understand the meanings of all those numbers, leading to the emergence of data literacy as the main means of deriving meaningful information from data (Bryla, 2018). In education, the development of new technology has prompted sweeping changes in teaching and learning, and has brought a new research perspective to teachers' literacy. In particular, data literacy is now considered a key aspect of teachers' professional development (TPD).

**Figure 1:**
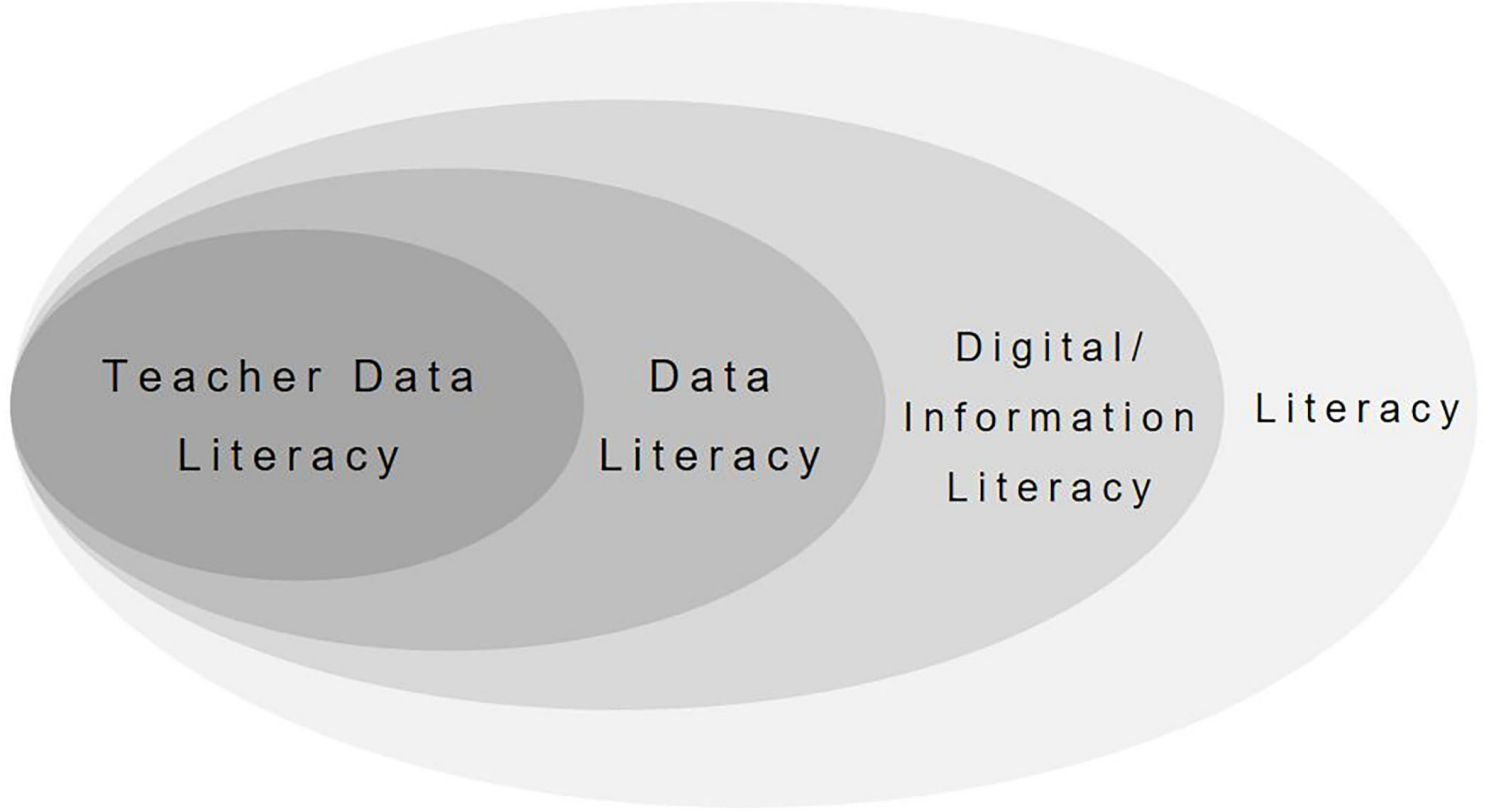
Related concepts of TDL.

Data literacy refers to “a set of abilities relating to the use of data” (Maycotte, 2014; Wolff et al., 2016), and teacher data literacy (TDL) is only one small form of it (see Figure 1). According to the Data Quality Campaign (2014), TDL can be defined as the process of continuously, effectively, and ethically accessing, interpreting, processing, and exchanging various types of data from state, local, classroom, and other sources to improve students' performance in a manner appropriate to the professional roles and responsibilities of educators. In an alternative definition, (Mandinach et al., 2015a) described TDL as an ability to collect, analyze, and interpret all kinds of educational data to help determine instructional measures. Overall, while no unified and comprehensive definition of teacher data literacy in the field of education currently exists, it is widely understood to combine an understanding of data, discipline, practice, pedagogy, and students (Mandinach et al., 2015a) to improve pedagogical decision-making (Ndukwe and Daniel, 2020). The assumption is that better data literacy will improve teachers' ability to instruct their students and will ultimately raise student performance levels (Mandinach and Gummer, 2016a). Moreover, a data-driven decision-making process will provide teachers with more accurate measures of pedagogy, assessment, and classroom management, and accelerate their professional development (Kennedy-Clark and Reimann, 2021). Recent studies have also stressed the need for teachers to develop their practical and communicative skills by sharing timely and targeted feedback data instantly with students (Cui et al., 2019). Along with teachers, school leaders are now advised to base interventions on data wherever possible (Kippers et al., 2018; Mandinach and Schildkamp, 2021), reflecting its power to transform education at all levels and in all aspects (Mandinach et al., 2015b; Wolff et al., 2016).

Emerging technologies and policy reforms both require data-driven educational decision-making (Geping et al., 2021). TDL plays a crucial role in improving teaching practice and promoting students' individual academic development. Steps toward achieving this goal are realized in several research frameworks (Marsh, 2012; Gummer and Mandinach, 2015; Maybee and Zilinski, 2015) that emphasize the importance of TDL to school principals, leaders, and learning assessors in terms of guiding more effective instruction (Cowie and Cooper, 2017). The growing abundance of school system data, such as electronic reports covering exam results, attendance, discipline, course participation, and course credits, provides data that guides school managers, teachers, and other stakeholders to improve the quality of education and teaching (Wayman et al., 2012). Therefore, TDL can transform data into operable and sustainable practices to support effective teaching, learning, and long-term professional development.

Unfortunately, previous studies show that teachers generally lack sufficient data literacy skills (Sun et al., 2016). Because inadequate data literacy knowledge can result in analytical misinterpretations with negative real-world consequences for learners (Ndukwe and Daniel, 2020), teachers require the knowledge to design evidence-based instruction and support learning (Mandinach et al., 2015b). Besides, the concept of data literacy remains relatively undefined (Papamitsiou et al., 2021), and further research is needed to understand how TDL requirements are linked to overall teachers' knowledge.

### Technological pedagogical content knowledge

Technological Pedagogical Content Knowledge (TPACK; Koehler and Mishra, 2006) is a widely used theory building on the earlier notion of pedagogical content knowledge (PCK). It identifies the three basic elements of teachers' knowledge as technology (T), subject content (C), and teaching pedagogy (Phillips and Harris, 2018). The complex interactions between these elements form four composite knowledge domains: PCK, TCK, TPK, and TPACK (Valtonen et al., 2017; Ifinedo et al., 2020). A change in any element of TPACK will cause alterations in the other elements, and improvements in any single element can help to develop teachers' knowledge (Chong, 2021). TPACK provides a fruitful way to resolve the many dilemmas teachers face when implementing educational technology in the classroom. It indicates that teachers must operate in the complex space of technology, content, and pedagogy, and integrate technology with traditional knowledge whenever possible (Cui and Zhang, 2021). By distinguishing between these three types of knowledge, TPACK can guide teachers to use specific technical tools (hardware, software applications, etc.) to help students better understand topics (Mishra and Koehler, 2006). Furthermore, TPACK raises teacher awareness of the need to integrate their knowledge of content, pedagogy, and technology, and many complex phenomena can be analyzed and explained within the framework (Jiawei and Zuhao, 2021). TPACK is therefore widely regarded as an effective analytic tool for improving teaching.

Yet despite the comprehensiveness of TPACK, it does not cover the teacher's digital competence in its entirety. While teachers have embraced smart technology (Zhang et al., 2020), a holistic conceptual framework is required to ensure teachers benefit in full against the backdrop of rapidly altering political, social, and economic conditions (Falloon, 2020). Such a framework should also acknowledge the widespread use of intelligent tools and platforms that can collect, store, visualize, and analyze educational data (Wayman et al., 2010). Therefore, teachers' knowledge should not only include technology (T), subject content (C), and pedagogy (P), but also data literacy (TDL), all of which influence the complex process of instruction. All of these elements are essential: without data, teachers may be unaware of the gap between students' current learning and their learning objectives, and may also be unable to narrow this gap without adequate pedagogical knowledge. As Datnow et al. (2021) claim, teachers who use data to pinpoint students' current states of knowledge and their difficulties can plan more effective and targeted teaching interventions. Diverse educational data expand the opportunities available to teachers to design effective lessons, and integrating TDL and TPACK will improve teachers' overall abilities.

Although TDL offers a powerful information literacy framework, teachers may struggle to implement it in the classroom (Pierce et al., 2014; Gelderblom et al., 2016; Filderman et al., 2021; Xin, 2021). Moreover, TDL has largely been excluded from accounts of teachers' knowledge development (Reeves and Chiang, 2018). Earlier teacher competence frameworks were useful for particular aspects of professional development (e.g., design, ICT, or related abilities), but could rarely accommodate emerging advances in educational data, or its usage (Papamitsiou et al., 2021). Marsh (2012) indicated the need for teachers to integrate data literacy with their professional knowledge. By integrating TDL with their general abilities, teachers can teach more effectively (Raffaghelli, 2019), and improve student outcomes (Conn et al., 2022).

## Materials and methods

### The ORID method

The objective–reflective–interpretive–decisional (ORID) method is a focus group method that generates dialogue about data to encourage effective decision-making and action (Wenhuei et al., 2021). ORID is used in the following ways (Hao, 2010): (1) revealing group reactions to certain issues or phenomena, (2) forming hypotheses and inferences about particular research through interviews, (3) improving and perfecting some quantitative research methods, and (4) explaining and elaborating on the results derived from other quantitative research methods. The purpose of this study was perfectly consistent with the second of these applications and was intended to develop a model of teachers' knowledge that integrates data literacy.

Brown (2019) noted that ORID requires objective, reflective, interpretive, and decisional questions to be asked, and these questions reflect four levels of critical thinking (Wooden, 1994). ORID reflects human internal cognition and promotes deeper thinking through effective team communications (Liu, 2019) while encouraging participants to shift from superficial to more in-depth reflection on specific topics (Ernst and Erickson, 2018). In ORID, a facilitator is usually needed to ensure the process includes the views of all, not just the voices of a dominant minority. This makes it easier for everyone to participate, starting from the objective facts about the topic, which enables participants to make scientific decisions. Furthermore, ORID facilitates faster qualitative data analysis and supports empirical reflection by encouraging participants to contribute regardless of their background (Fritzen-Pedicini et al., 2019). ORID has been used to generate data in a variety of sectors including the business, academic, and public/non-profit sectors. It has been adapted to face-to-face, phone-, and internet-based situations with children and adults. It is generally used with a small number of participants (usually 8–12, but fewer in academic or telephone focus groups).

### ORID participants

The study participants were screened using several criteria. First, participants needed either to understand PCK- or TPACK-related theories or to have participated in similar studies of academic theory construction. Second, they were required to have undertaken teaching practice activities in primary or secondary schools or to have watched a minimum number of similar teaching videos. Third, they needed to have engaged in teacher professional development (TPD) research, or have achieved relevant successes in their academic activities. The researchers evaluated whether participants met these criteria based on their feedback. Ultimately, 11 participants were recruited, including one professor (PRO1), three PhD students (PHD1-PHD3), five master's students (MAS1-MAS5), and two middle school teachers (MID1-MID2), all based in Changchun, Jilin province, China. The participants (two males and nine females) were aged between 23 and 43 years old. They discussed the Research Topic, and expressed their opinions on the questions, following the ORID method (see Table 1 for additional information).

**Table 1:** Demographic statistics of the participants in TDL-TPACK construction.

**Category**	**Code**	**Gender**	**Age**	**Rule 1**	**Rule 2**	**Rule 3**
Professor	PRO1	Male	43	√	√	√
PhD degree students	PHD1	Female	28	√	√	√
	PHD2	Female	27	√		√
	PHD3	Male	26	√	√	
Master degree students	MAS1	Female	23		√	√
	MAS2	Female	23	√		
	MAS3	Female	25	√	√	
	MAS4	Female	25	√		√
	MAS5	Female	24	√		√
Middle school teachers	MID1	Female	25	√	√	
	MID2	Female	26		√	

### Procedure

The TDL-TPACK framework had to be evidence-based so ORID was used to ensure the analysis was reliable. We appointed a facilitator to chair the interviews, overcome any unwillingness from the participants to share their views (Ernst and Erickson, 2018), and generate positive outcomes. First, we drew up a question list based on the four-level ORID questions, and the participants were selected based on the three criteria above. The participants were then interviewed based on the ORID process (see questions in Table 2). At this stage, the facilitator asked each participant to respond to the four-level questions one by one, while other participants contributed and discussed their views to deepen their understanding of the issues. Third, the facilitator summarized the possible links between TDL and TPACK, forming several hypothetical frameworks after further discussion. Following a further round of reflective discussion (see Table 2), the TDL-TPACK framework was finalized.

**Table 2:** Questions for participants.

**Levels**	**Questions**	**Purpose**
Objective	O1: Do you know the theory that is related to teacher knowledge	Discover participants' objective views on the teacher knowledge and data literacy.
	O2: What are the necessary components of teacher knowledge?	
	O3: What's your understanding of teacher data literacy?	
Reflective	R1: What's your feeling about teacher daily instruction with data?	Discover participants' reflective feelings on integrating TDL and TPACK.
	R2: How do you feel about integrating teacher data literacy and TPACK (an integration model of technology, content, and pedagogy)?	
Interpretive	I1: What can you learn from the smart learning environment when forming your own teacher data literacy?	Discover the significance and inspiration of teacher data literacy.
	I2: What's your opinion on the relationship between teacher data literacy and teachers' knowledge structure?	
Decisional	D1: How will you build the TDL-TPACK framework considering the updating technology, content, and pedagogy?	Make decisions on the construction of TDL-TPACK.
	D2: Please explain why you want to build a framework like this?	
Another reflective	A1: Do you think these teacher knowledge frameworks' construction is reasonable?	Measure whether the TDL-TPACK construction is effective and reliable.
	A2: What other changes are needed in TDL-TPACK when considering other influencing factors?	

### Construction of the framework

At the objective stage of the process, three questions were asked to discover the participants' views of teachers' knowledge and data literacy. In general, the postgraduate degree students were more familiar with this issue and their comments were more closely aligned with standard PCK or TPACK theory (O1). Although the middle school teachers were unaware of such theories, they also emphasized the different components of teachers' knowledge, such as teaching methods, subject content knowledge, class management, technology, etc. (O2). However, most participants, such as PHD3, MAS1, MAS4, and MID2, could not describe the typical components of teacher data literacy (O1).

The reflective stage consisted of two questions to generate ideas about integrating TDL and TPACK. While the participants were unaware of TDL *per se*, they articulated the considerable positive value of data to teaching and learning (R1). These viewpoints reflected the transformation of traditional teaching methods *via* the growth of smart technologies. The participants expressed the urgent importance of integrating teachers' data literacy and knowledge (R2): they perceived that technology would enable them to access and analyze educational data such as teaching and learning performances.

At the interpretive stage, two questions were asked about the significance of teacher data literacy. The participants considered that accessing, processing, and interpreting educational data was highly important (I1), although many lacked the appropriate skills. For example, MID1 and MID2 stated they did not know where to obtain relevant learning and teaching data. Relying on their own abilities made it difficult for teachers to process, interpret, and apply educational data to guide their instruction. These results point to the need to systematically link data skills and knowledge to the knowledge structure of teachers to improve practice and professional development (I2).

**Figure 2:**
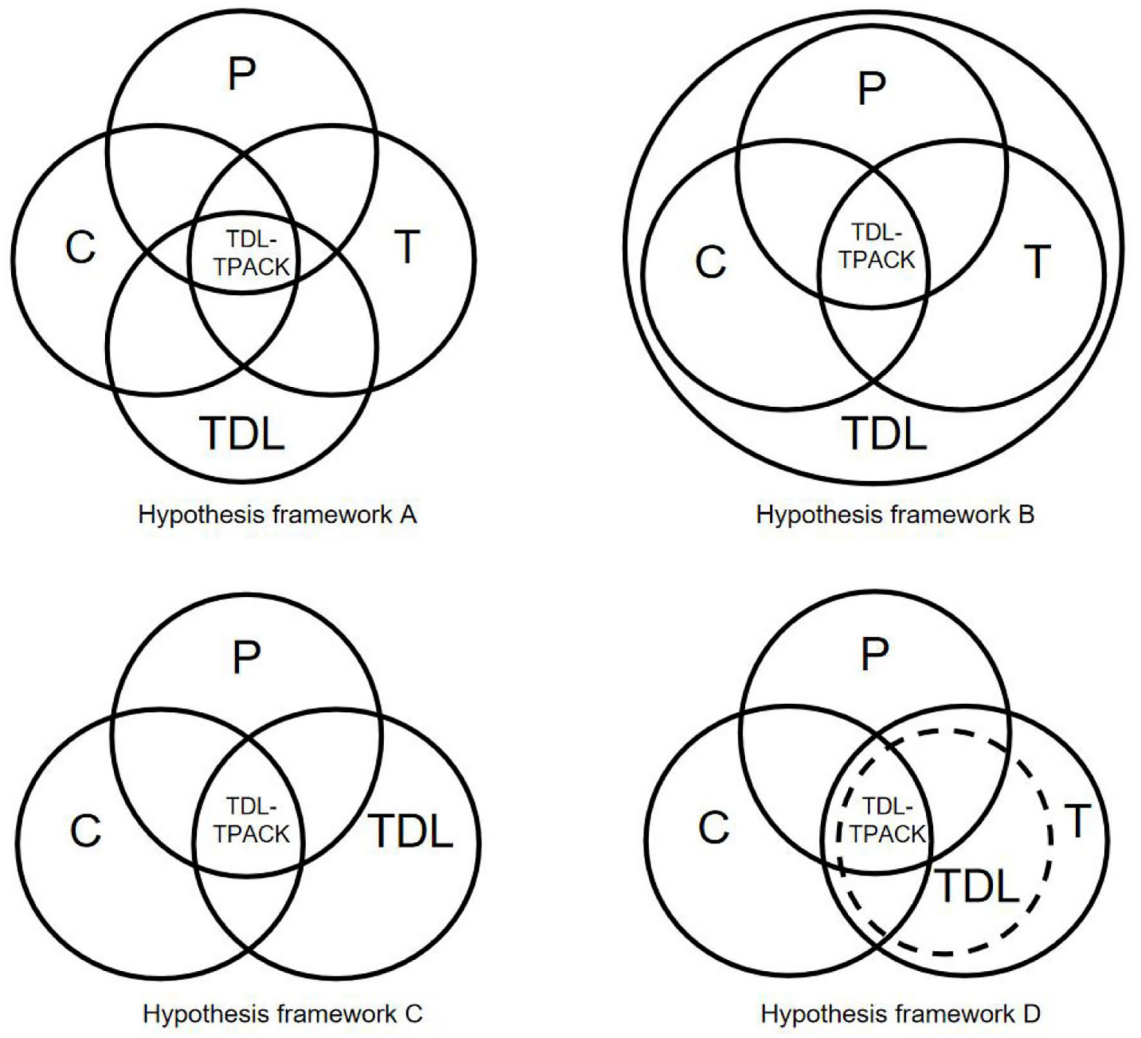
Different types of hypothesis frameworks of TDL-TPACK based on ORID. Hypothesis framework A—TDL is considered as a separate domain of teacher knowledge. In other words, the TDL is seen as an independent component, and is connected with the original three components of TPACK. In this perspective, proponents argue that teachers should think about and take instructional design in the context of a complex interaction of four components and the fusion of these four components constitutes the core knowledge field of teachers. Hypothesis framework B—TDL is considered as a contextual component of the original three components of TPACK, and it offers a fundament and background for the integration of TDL-TPACK. Proponents of this view believe that teachers should integrate pedagogy, technology, and content in a way that takes full account of teacher data literacy. Hypothesis framework C—TDL replaces the original technology knowledge (TK) and integrates it with pedagogy knowledge (PK) and content knowledge (CK), resulting in a core knowledge field of teachers. The proponents of this idea argue that the changing of technology leads to the creation of data literacy, and therefore teacher data literacy should replace basic knowledge of technology for the development of the smart education era. Hypothesis framework D—TDL is seen as an important component of TK, and it integrates with PK and CK, resulting in an core knowledge field of teachers. Proponents of this framework emphasize that the role and breadth of technology is broader than TDL. Whereas TDL builds on traditional technology, it is as an intrinsic dimension of TK that TDL can only be integrated with other elements of the TPACK.

Finally, at the decisional stage, the participants were asked two questions concerning the construction of the proposed TDL-TPACK framework. After a detailed discussion with participants, several different frameworks were proposed (Figure 2), taking into account the impact of data literacy on the traditional TPACK framework (D1). TDL occupied different positions among the TPACK elements, and its effects on traditional technology, subject content, and pedagogical approaches therefore differed.

**Figure 3:**
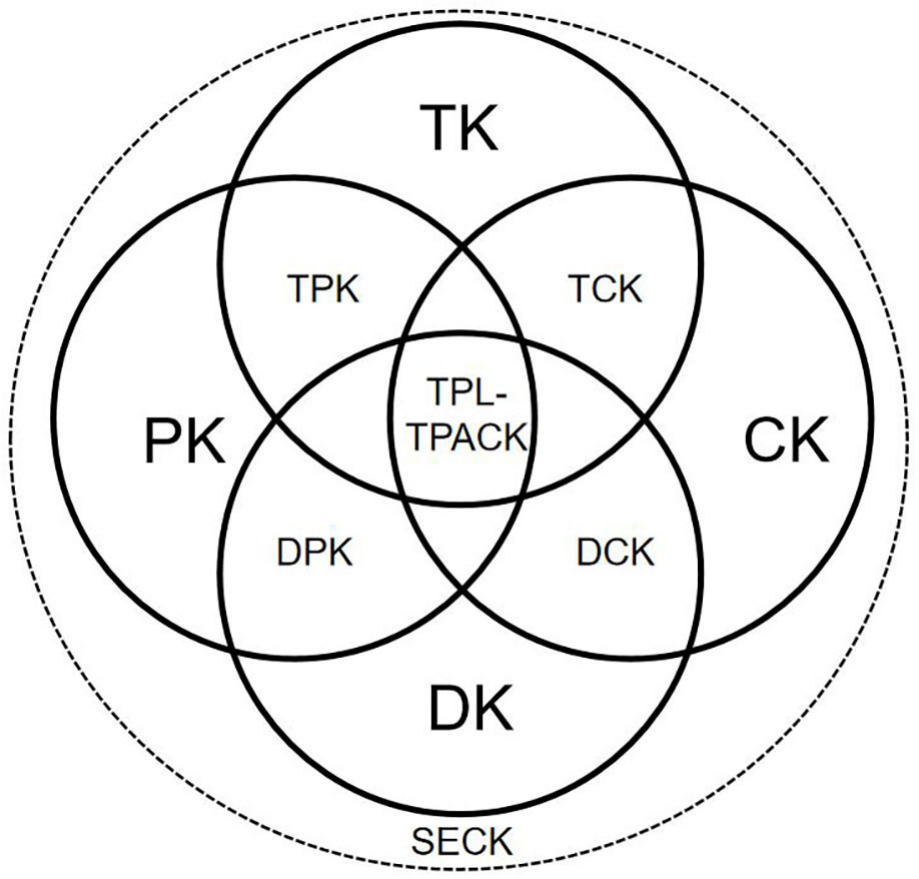
TDL-TPACK framework for teachers.

Although the proposed frameworks positioned teacher data literacy differently, the participants shared the view that technology, content, and pedagogy needed to be integrated with data literacy to promote the professional development of teachers. The four hypothesized frameworks were built from the analyzed interview data of different participants. To assess the effectiveness of these frameworks and develop a common view of the TDL-TPACK framework, we initiated another reflective session for the participants. After further interviews, most of the participants agreed that their perspectives were effectively represented by the four hypothesized frameworks (A–D) and that the teachers' knowledge framework was configured reasonably well (A1). However, they also stated that while the frameworks acknowledged the importance of TDL, they failed to account for the intrinsic link between technology and data literacy because TDL is broader in scope and may itself already encompass teachers' knowledge related to technology, pedagogy, and subject content. The participants added that simply combining teacher data literacy (TDL) with technology (T), pedagogy (P), and content (C) was insufficient. Hence, after further deep discussion, it was decided the framework should integrate teacher data literacy based on the knowledge of data (DK), pedagogy (PK), subject content (CK), and technology (TK). Because TDL is related to pedagogy, technology, and subject knowledge, data knowledge (DK) was treated as a separate category. Moreover, the importance of the smart learning process needed to be acknowledged given the pace of technological change, so a smart education context (SEC) was added to the final teachers' knowledge framework (Figure 3) that acknowledged other influencing factors in TDL-TPACK (A2). The SEC element emphasized the need for teachers to navigate the smart educational environment and adapt to its emerging features, further developing their knowledge and professional development.

## Framework application

### Questionnaire

Teachers' knowledge is a complex process, and different research perspectives are reflected in different measurement approaches. In fact, there are at least two perspectives on teacher professional knowledge: a situated and a cognitive one, and the latter is usually measured quantitatively (Evens et al., 2018). In general, there are many different ways of measuring teachers' knowledge, such as questionnaires, interviews, classroom observations, etc., and self-report questionnaires are in widespread use for this purpose (Koehler et al., 2012). They apply to large-scale research, produce more generalizable results (Stahnke et al., 2016), and are more effective and reliable than other research methods (Voogt et al., 2013). To measure teachers' knowledge against the new TDL-TPACK framework, three measurement scales were used: the first was TPACK.xs, developed by Schmid et al. (2020), the second was based on a teacher data literacy scale developed by Xin and Xianmin (2020), and the last used Teacher's Precision Teaching Ability Model (Jian, 2020), which focuses on the competencies and knowledge related to precision teaching in the “big data” era. The final questionnaire contained 13 dimensions and 50 items that demonstrated great reliability and validity, as shown in Table 3. Example items are provided below.

**Table 3:** TDL-TPACK framework for teachers.

**Dimensions**		**Items**	**Cronbach's alpha**	**Source**	**Example**
PK	Pedagogical knowledge	4	0.91	Schmid et al., 2020	I can use all kinds of teaching methods in class.
CK	Content knowledge	4	0.92		I have enough knowledge about my teaching topic and subject.
TK	Technological knowledge	4	0.90		I have the skills in using technology.
DK	Data knowledge	4	0.92	Jian, 2020; Xin and Xianmin, 2020	I have basic knowledge related to educational data (such as source, characteristics, and value).
SECK	Smarting education contexts knowledge	3	0.92	/	I can tell which devices constitute the smart education environment.
PCK	Pedagogical content knowledge	4	0.93	Schmid et al., 2020	I know how to choose effective teaching methods to guide students' thinking and learning.
TPK	Technological pedagogical knowledge	4	0.88		I can apply the technology to different teaching activities.
TCK	Technological content knowledge	4	0.91		I know which techniques should be used in my teaching content.
TPCK	Technological pedagogical content knowledge	4	0.93		I can appropriately combine teaching content, technology and teaching methods to instruct student.
DPK	Data based pedagogical knowledge	4	0.95	Jian, 2020	I have the ability to process and analyze educational data, and can interpret data visually.
DCK	Data based content knowledge	3	0.93	Xin and Xianmin, 2020	I know some data platform and data source related to my subject teaching.
DTK	Data based technological knowledge	3	0.92		I can use technology to visualize educational big data.
TDL-TPACK	Teacher data literacy based technological pedagogical content knowledge	5	0.95	Xin and Xianmin, 2020	I can adjust teaching behavior and teaching methods according to the results of data processing.
Total	/	50	0.97	/	/

### TDL-TPACK survey respondents

After adding questions related to basic teacher and school information, the self-report questionnaire was uploaded to the Questionnaire Star platform (https://www.wjx.cn/), where it remained open between March and April 2022. A total of 696 valid questionnaires were obtained from the respondents, who were 17.2% male and 82.8% female. In terms of age distribution, 72.4% of the sample was aged 20–30, while other age groups accounted for 27.6% of responses. Most participants had certain ages for teaching while 9.8% of respondents had 5–10 years of professional teaching experience. Further details about academic qualifications, titles, and grades can be found in Table 4.

**Table 4:** Demographic statistics of the participants in TDL-TPACK applying.

		**Number**	**Accumulative percent%**			**Number**	**Accumulative percent%**
Gender	Male	120	17.2	Academic qualifications	Below Bachelor's degree	24	3.4
	Female	576	100		Bachelor's degree	308	47.7
Age	Below 25	60	8.6		Masters' degree	340	96.6
	20–30	504	81		Doctoral degree or above	24	100
	30–40	92	94.3	Title	Level 3	148	21.3
	40–50	24	97.7		Level 2	408	79.9
	Over 50	16	100		Level 1	100	94.2
Teaching age	0–5	552	79.3		Senior or above	40	100
	5–10	68	89.1	Grade	Primary 1–3	120	17.2
	10–20	36	94.3		Primary 4–6	116	33.9
	20–30	12	96		Secondary	320	79.9
	Over 30	28	100		High school	140	100

### Data analysis

The descriptive statistical analysis of pedagogical content knowledge integrating teachers' data literacy (hereafter referred to as teachers' knowledge) displayed high means and small standard deviations in the foundational dimensions of PK, CK, TK, DK, and SECK. This was also true of scores for the various composite knowledge dimensions formed from the foundational dimensions (the lowest of these was SECK: *M* = 4.02; *SD* = 0.03, as shown in Table 5). All the dimensions scored above 4 (somewhat consistent), with most teachers self-reporting high literacy performance in the dimensions of technology, pedagogical content, pedagogy, the smart educational environment, and data knowledge. The three highest performance dimensions of teachers' knowledge were related to the content, pedagogy, and subject pedagogical knowledge integrating technology (TPCK), while the lowest were the smart teaching environment (SECK), technology, and data-related dimensions of teachers' knowledge. The low SECK means indicated that some teachers were unfamiliar with the relevant components and typical technologies of intelligent teaching environments.

**Table 5:** Demographic statistics for TDL-TPACK.

	**Mean**		**Std. deviation**	**Variance**	**Skewness**		**Kurtosis**	
	**Statistic**	**Std. error**	**Statistic**	**Statistic**	**Statistic**	**Std. error**	**Statistic**	**Std. error**
PK	4.24	0.03	0.79	0.63	−1.73	0.09	4.42	0.19
CK	4.31	0.03	0.75	0.56	−2.08	0.09	6.77	0.19
TK	4.07	0.03	0.79	0.63	−1.04	0.09	1.39	0.19
DK	4.08	0.03	0.83	0.69	−1.26	0.09	2.20	0.19
SECK	4.02	0.03	0.83	0.69	−1.25	0.09	2.33	0.19
PCK	4.29	0.03	0.68	0.46	−1.66	0.09	5.62	0.19
TPK	4.17	0.03	0.71	0.51	−1.25	0.09	3.31	0.19
TCK	4.20	0.03	0.74	0.55	−1.37	0.09	3.28	0.19
TPCK	4.24	0.03	0.70	0.48	−1.59	0.09	4.84	0.19
DPK	4.07	0.03	0.80	0.64	−1.29	0.09	2.29	0.19
DCK	4.15	0.03	0.73	0.54	−1.39	0.09	3.51	0.19
DTK	4.13	0.03	0.75	0.56	−1.35	0.09	3.02	0.19
TDL_TPACK	4.18	0.03	0.73	0.54	−1.42	0.09	3.67	0.19

Non-parametric gender tests of the 13 dimensions of TPACK using the Mann-Whitney U-test indicated that the content knowledge (CK), technical knowledge (TK), and technical pedagogical knowledge (TPK) dimensions showed no major differences (*p* > 0.05) between male and female teachers. However, significant differences existed between male and female teachers on the other 10 dimensions of TDL-TPACK (see Table 6).

**Table 6:** Mann–Whitney *U* test for TDL-TPACK in gender.

**Null hypothesis**	**Sig**.	**Decision**
The distribution of PK is the same across categories of gender	0.001	Reject
The distribution of CK is the same across categories of gender	0.063	Retain
The distribution of TK is the same across categories of gender	0.926	Retain
The distribution of DK is the same across categories of gender	0.000	Reject
The distribution of SECK is the same across categories of gender	0.016	Reject
The distribution of PCK is the same across categories of gender	0.024	Reject
The distribution of TPK is the same across categories of gender	0.058	Retain
The distribution of TCK is the same across categories of gender	0.039	Reject
The distribution of TPCK is the same across categories of gender	0.007	Reject
The distribution of DPK is the same across categories of gender	0.001	Reject
The distribution of DCK is the same across categories of gender	0.003	Reject
The distribution of DTK is the same across categories of gender	0.001	Reject
The distribution of TDL-TPACK is the same across categories of gender	0.039	Reject

**Figure 4:**
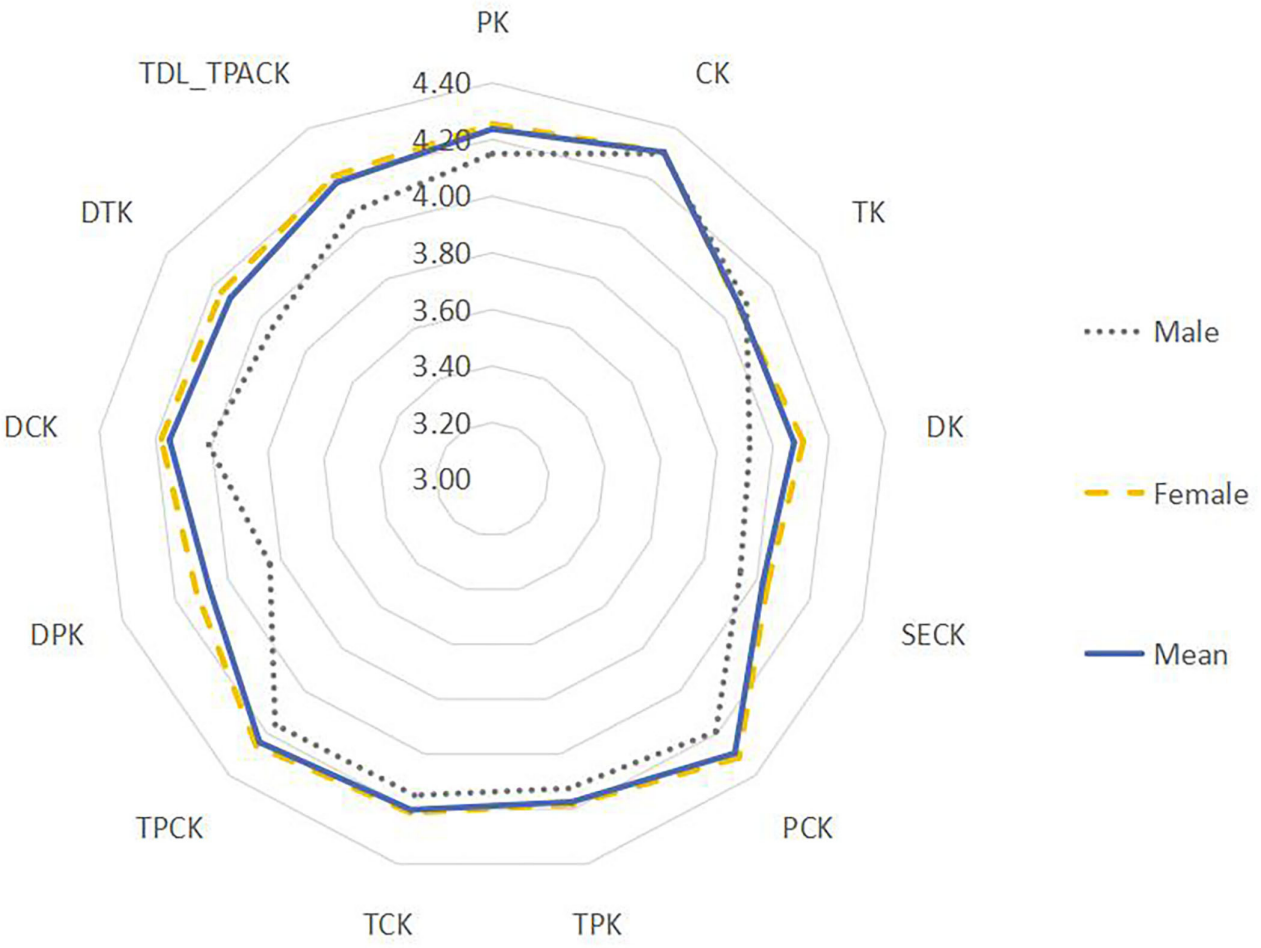
Gender difference.

Figure 4 displays the radar chart of teachers' knowledge based on descriptive statistics, showing that the performance levels for most dimensions of TDL-TPACK differed significantly by gender. Although the scores of male and female teachers resembled each other, with mean scores around 4 and low standard deviations, female teachers scored higher on knowledge performance in the TDL-TPACK. This indicated that female teachers were better able to integrate teacher data literacy with TPACK, while male teachers were slightly weaker than women overall.

**Figure 5:**
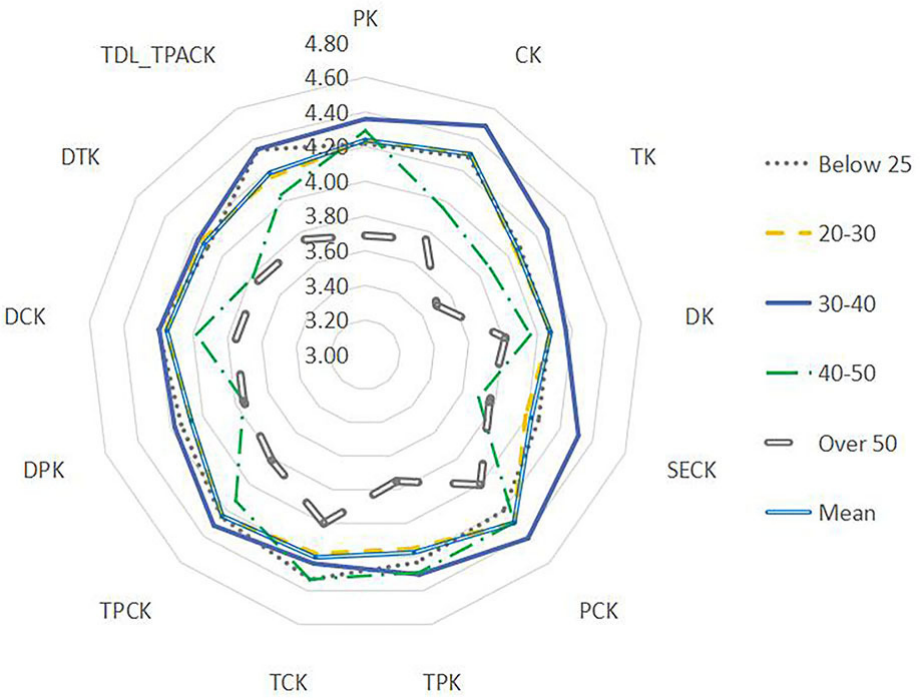
Age difference.

**Figure 6:**
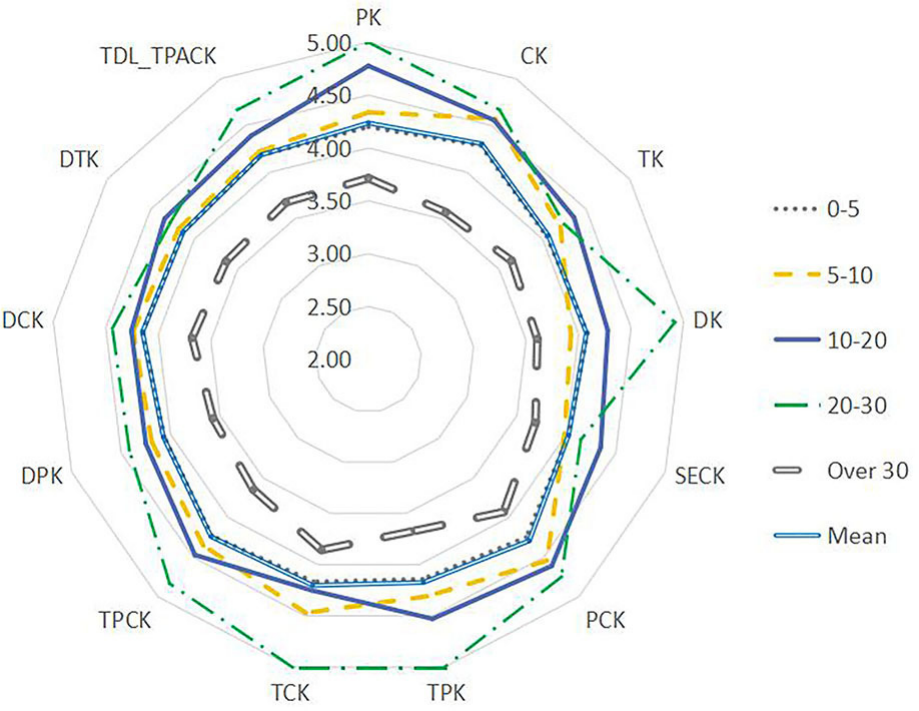
Teaching age difference.

**Figure 7:**
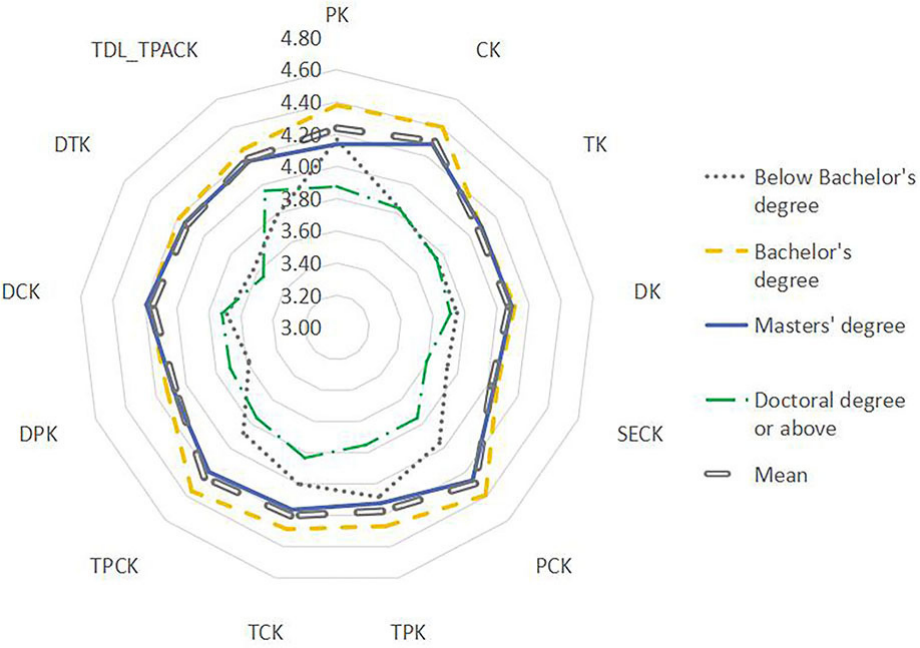
Academic qualifications difference.

**Figure 8:**
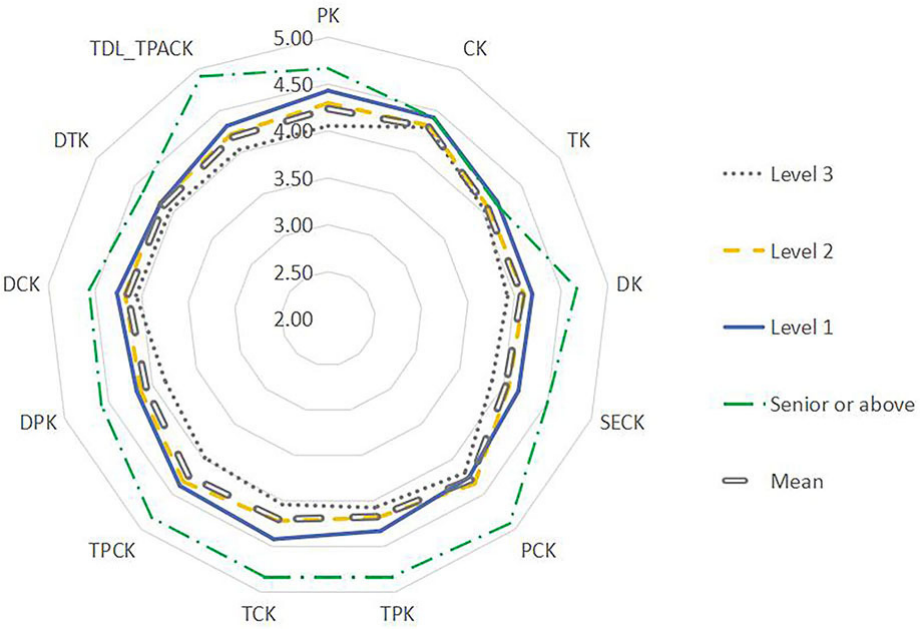
Title difference.

**Figure 9:**
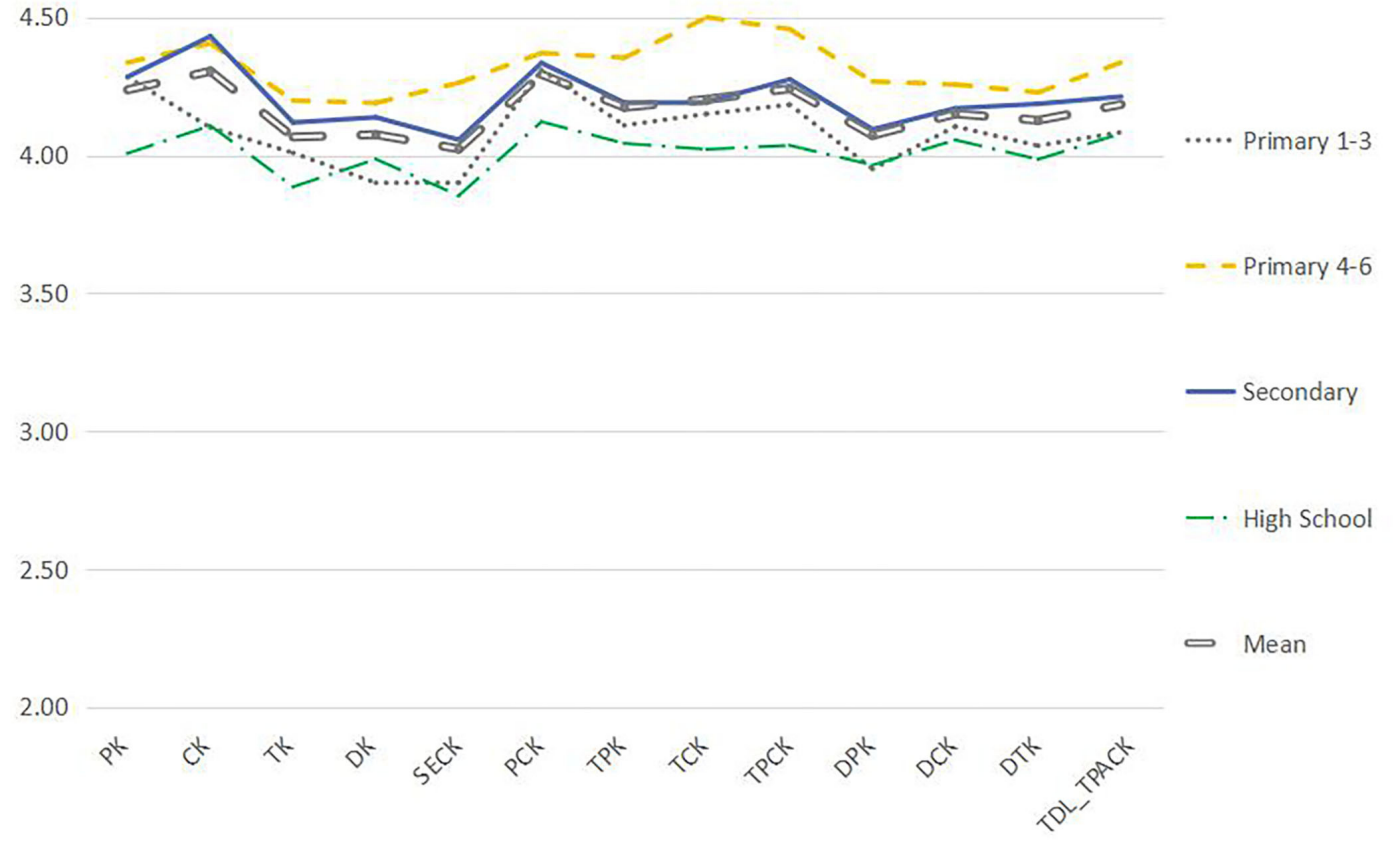
Grade difference.

Using the Mann–Whitney *U*-test, the data were analyzed by teachers' age, length of service, academic qualifications, title, etc. (see Figures 5–**9**). There were five findings of particular significance. First, teachers aged 30–40 had higher levels of knowledge and outperformed teachers in other classifications, while teachers aged more than 50 expressed the lowest levels of knowledge, as shown in Figure 5. Second, teachers with 20–30 years of service had higher levels of knowledge, while those with more than 30 years of teaching experience were weaker overall (Figure 6). Third, teachers with bachelor's and master's degrees performed better, while teachers at other levels were slightly weaker overall (see Figure 7). Fourth, the TDL-TPACK performances were positively correlated with teacher seniority as represented by job title, with teachers at the senior level or above performing more strongly than those working at levels 1–3, as shown in Figure 8. Finally, we found that the grade taught by teachers was not a core element of TDL-TPACK, although teachers of the upper elementary grades (i.e., primary 4–6) may perform better, as shown in Figure 9.

## Discussion

The ORID discussions indicated that many participants attended closely to the impact of emerging technological changes on the educational process. Recent research has also highlighted the important role technology plays in teachers' knowledge systems. Educational data encourage teachers to reflect on their daily teaching and some previous data-based teacher literacy frameworks emphasize that teachers should strive to integrate data into their professional practice. Although most teachers are aware that data literacy leads to better decision-making (Kennedy-Clark and Reimann, 2021), many are unable to integrate technology into their classroom practice and some may refuse to do so altogether. One reason for this may be that technology requires teachers to constantly innovate approaches and shift their “comfort zone” away from classical or conventional teaching, which many resist doing (Agustini et al., 2019). Hence, a more comprehensive framework for teachers' professional development that incorporates TDL is required.

This research emphasizes that data literacy must be integrated with teachers' traditional knowledge of technology, pedagogy, and subject content. We identified the relationships between several different components of the TDL-TPACK framework: pedagogic knowledge (PK), subject content knowledge, technological knowledge (TK), smart educational context knowledge (SECK), and teachers' data knowledge (DK). Using ORID, we confirmed that DK was viewed as an important component of core teachers' knowledge alongside PK, CK, TK, and SECK. This implies the need to embed TK, PK, and CK into smart learning environments, with key educational decisions ultimately becoming data-driven. This framework prompted greater awareness that teachers' knowledge of smart learning would lead to increased data literacy and expand into disciplinary pedagogical knowledge that integrates TDL.

We then used surveys to investigate how teachers assessed their own knowledge against the TDL-TPACK framework. The results indicated that performances were highest among experienced teachers in their 30s and 40s, but diminished incrementally among teachers aged over 50, despite their greater teaching experience. These findings corroborate those of Ifinedo et al. (2020), who reported that teaching experience and ICT skills were negatively correlated. A more advanced educational background does not entail the ability to combine teacher data literacy with TPACK more effectively, but this knowledge was positively associated with the title or role of teachers. Although teachers with higher academic qualifications and certification backgrounds exhibited relatively weak levels of knowledge in the questionnaire, they might do better in classes where students achieve at higher levels and are less likely to drop out (Darling-Hammond, 2000). Wang et al. (2021) also noted that teachers with more advanced educational backgrounds may experience fewer difficulties with course content.

High means and small standard deviations were recorded for all 13 of the survey's dimensions (the overall mean was >4), reflecting consistently high levels of self-reported knowledge: most teachers perceived themselves to be highly literate in the dimensions of technology, content, pedagogy, intelligent educational environment, and data knowledge. However, as shown in Table 5, the three highest performing dimensions of teachers' knowledge were those concerned with content (CK), pedagogy (PK), and subject pedagogical knowledge integrating technology (TPCK), while the lower-scoring dimensions were those related to knowledge of the smart educational context (SECK), technology (TK), and data (DK). This confirms the findings of Taimalu and Luik (2019) that technology is incompatible with some teachers' disciplines and teaching approaches, even when its potential practical value is recognized. Moreover, the finding was that qualified teachers aged 30–40 were more knowledgeable and performed better than all other age groups, particularly the 50-plus group. While experienced teachers may have more content and pedagogic knowledge, they may also be untrained, more resistant to using educational ICT applications (Yilmaz and Bayraktar, 2014; Saltan and Arslan, 2017), and therefore less technologically skilled and knowledgeable.

This research also has positive implications for participants. The study encouraged a range of stakeholders (teachers, students, professors, etc.) to contribute to a specific framework for teachers to take different voices into account. The ORID process supports the discovery of knowledge by enabling individuals to attribute meanings to their actions *via* the “looking-glass self” formed by observing and interacting with others (Stevanovic and Weiste, 2017). In other words, the collective ORID discussion benefitted educational practitioners by stimulating more profound reflections on the role of technology in teaching, enabling the TDL-TPACK model to be constructed and refined. Although the teachers acknowledged they did not meet all the standards, their integrated model of teachers' data literacy pointed to their future professional development efforts. The implications are not only positive for long-term teaching provision but also for student learning and enriching educational policy, curricula, and institutions (Liu and Zhang, 2021). The hybrid research methods used in this research enabled teachers' knowledge and their data literacy to be integrated, leading to the discovery of significant factors and cognitive processes, and ultimately, a consensus view. Such skills- or knowledge-based conceptual framework could initiate teacher-led curricular changes (Henderson and Corry, 2020). Teachers should take opportunities to enhance their self-efficacy and confidence in technology and data applications; a positive attitude from both teachers and school leaders to blending technology, experience, and pedagogical content knowledge will help improve practice across the academy (Rohaan et al., 2012).

## Conclusion

This research began by reviewing research into teacher data literacy and the theory of TPACK. Using the qualitative ORID method, we built a teacher data literacy integrated knowledge framework (TDL-TPACK) relevant to smart learning environments. Together with other teachers, we identified and reviewed four potential frameworks of teacher data literacy integrated with specific elements of TPACK. Despite the different positions of TDL in these frameworks, it is widely understood that teacher data literacy must be integrated into a comprehensive teachers' knowledge framework that includes technology, pedagogy, and content. ICT is also key to integrating teachers' knowledge, skills, and subject areas (Heitink et al., 2016; Ifinedo et al., 2020). We found that data literacy may cover both technical and pedagogical attributes, and therefore, data knowledge (DK) must be integrated with teachers' technological pedagogical content knowledge to produce a new knowledge framework. The research culminated in a common view that TDL is an important dimension of core teachers' knowledge. This process of integration also requires an understanding of smart education contexts (SECK).

In the quantitative phase of this mixed-methods study, we applied the updated TDL-TPACK frame to explore the current state of teachers' knowledge. The framework was found to be useful in exploring teachers' knowledge structures, based on descriptive statistics, radar plots, and Mann-Whitney U-tests, which demonstrated that teachers' knowledge varied according to several factors. For example, the study found that females performed better than male teachers, that mature teachers in their 30s and 40s may have performed better than others and that teachers' knowledge of TDL-TPACK was positively correlated with their job role or title. The results point to the need to tailor support for teachers' professional development from the perspective of integrating teacher data literacy with TPACK, according to these factors. We also note that technological knowledge is conceptually broader than data literacy itself, and educational data can be generated and used in technology-based environments, such as Smart Education Context (SEC). This means a teacher must first be able to understand and use the underlying technologies, and then be able to understand the data they generate, even as new technologies emerge. We further recommend that teachers closely attend to student-centered teaching methods and remain aware of the impacts of emerging technology, tools, and platforms in SEC environments. This will facilitate and optimize the presentation and acquisition of teaching content and assist the development of learning resources, students, and teachers themselves (Wang et al., 2022).

Overall, the study has highlighted the important role of teachers' knowledge in the formation of data literacy and the need to take an integrative perspective on classroom instruction. In particular, it has emphasized the need to consider how teachers develop data literacy when using pedagogy that integrates technology. Further investment in teacher training programs is encouraged to help teachers integrate data literacy, PCK, or TPACK knowledge to improve their classroom practice (Miller-Bains et al., 2022). Governments, schools, and universities can carry out targeted teacher professional development activities or training programs according to the TDL-TPACK framework, helping pre- or in-service teachers achieve this goal in the future. For example, teachers can be trained using pre- or post-test data to design more effective teaching interventions (Kippers et al., 2018). Further findings can promote teacher training programs in online, offline, or hybrid learning environments that target teachers' data literacy and TPACK-related skills. It is important to sustain such programs or capacity-building activities throughout teachers' careers, and technical assistance should be provided for better professional development (Mandinach and Jimerson, 2021). This training should first encourage teachers to attend to the formation and development of the five essential knowledge components (TK, PK, CK, DK, and SECK). Teachers should then strive to integrate these to form a compound knowledge that can subsequently be tested. The core element of teachers' knowledge, TDL-TPACK, should also be formed through practical involvement in long-term teacher training projects. In brief, the TDL-TPACK framework we present here can provide a solid foundation for in-service teachers' knowledge, literacy, and skills testing, thereby supporting comprehensive professional development in the context of smart education.

## Limitations and future works

In this study, ORID, a focus group method, was used to form a teachers' knowledge framework. However, as an interview analysis method, ORID cannot be applied to all social science research. As stated by Lewis-Beck et al. (2004), the effectiveness of this method deserves further consideration in making statistical inferences, considering its small number of participants. Self-reported questionnaires were used to discover the actual state of teachers' knowledge. yet the participants still need to be further expanded, i.e., stratified sampling in different economic development levels of districts (Dong et al., 2020), to further enrich the framework. The TDL-TPACK framework also needs to be supported by deep quantitative analysis to explore the potential relationships between specific inner elements. The factors affecting the establishment of TDL-TPACK also need to be systematically considered. Besides, an evidence-based framework construction process is encouraged to be further carried out, and a controlled experiment or action research method should be conducted to ensure its reliability. Further studies should also be considered to validate and improve teachers' data use and how to enhance the integration of teachers' data literacy and TPACK to promote long-term teachers' professional development.

## Data availability statement

The original contributions presented in the study are included in the article/Supplementary material, further inquiries can be directed to the corresponding author/s.

## Ethics statement

The studies involving human participants were reviewed and approved by the Institute of Educational Technology of Northeast Normal University. Written informed consent to participate in this study was provided by the participants.

## Author contributions

YC contributed to conception, design, and performed the statistical analysis. HZ edited initial manuscript and conducted project approval the manuscript. Both authors contributed to manuscript revision, read, and approved the submitted version.

## Funding

This work was supported by the project of Jilin Provincial Development and Reform Commission Jilin Engineering Research Center of Integration and Innovation of Education and Artificial Intelligence (Grant No. 2019694), the project of Jilin Provincial Science and Technology Department Jilin Province Cross-regional Cooperation Science and Technology Innovation Center of Education and Artificial Intelligence (Grant No. 20200602015ZP), the open project of Intelligent Education of the State Key Laboratory of Cognitive Intelligence Research on the practice of smart classroom precision teaching in frontier rural primary and secondary schools (Grant No. IED2021-Z001), the project of Undergraduate Higher Education Teaching Reform in Jilin Province Research on the innovative training mode of excellent journalism and communication talents in the smart environment (Grant No. JLJY202118470787), and the project of Creative Education in promoting the teaching mode reform of normal students' subject understanding by Northeast Normal University Research on the cultivation and development of normal students driven by big data (Grant No. 21CZ0011).

## Conflict of interest

The authors declare that the research was conducted in the absence of any commercial or financial relationships that could be construed as a potential conflict of interest.

## Publisher's note

All claims expressed in this article are solely those of the authors and do not necessarily represent those of their affiliated organizations, or those of the publisher, the editors and the reviewers. Any product that may be evaluated in this article, or claim that may be made by its manufacturer, is not guaranteed or endorsed by the publisher.
